# The Role of Clinician Training in Translating Clinical Decision-Making into Timely VHA-Guided Care. Comment on Natalini et al. Viscoelastic Hemostatic Assays in the Management of Trauma-Induced Coagulopathy: A Clinical Update. *J. Clin. Med.* 2026, *15*, 12

**DOI:** 10.3390/jcm15176555

**Published:** 2026-08-25

**Authors:** Jadon Kind, Kaitlyn Kulesus

**Affiliations:** 1 College of Osteopathic Medicine, Kansas City University, Kansas City, MO 64106, USA; jadon.kind@outlook.com; 2 Department of Anesthesiology, University of Oklahoma Health Sciences Center, Oklahoma City, OK 73104, USA

We read with great interest the recent narrative review by Natalini et al., which provides a comprehensive overview of the role of viscoelastic hemostatic assays (VHAs) in trauma-induced coagulopathy (TIC) [1]. The authors highlight several physiologic advantages of VHAs over conventional coagulation tests (CCTs), including their ability to provide dynamic, whole-blood assessment and reduce blood product utilization. They also appropriately acknowledge important real-world considerations, including the need for clinical training and the development of center-specific treatment algorithms to aid efforts in improving VHA reproducibility. Furthermore, VHAs are described as enabling rapid clinical decision-making due to their point-of-care availability and reduced turnaround time compared to CCTs. While the authors appropriately identify clinician training as a potential limitation to VHA implementation, the implications of this requirement in time-sensitive, trauma-induced coagulopathy warrant more detail and further consideration.

In such settings, the clinical utility of VHAs depends not only on the speed of result generation, but also on the efficiency with which those results can be interpreted and translated into therapeutic action. Interpretation of VHA results requires integration of multiple parameters reflecting distinct phases of coagulation, often within algorithm-based pathways [2]. As VHAs are variably implemented and algorithms between institutions vary, this process relies heavily on clinician familiarity and training [3]. As a result, interpretation may not be instantaneous, particularly in high-acuity settings where rapid and confident decision-making is essential.

Recent studies have demonstrated variability between expert clinical interpretation of VHA results and institution-specific algorithmic frameworks [4]. In addition, a survey from a hospital in Shanghai found substantial gaps in clinician understanding of VHAs, with many respondents expressing limited familiarity and negative perceptions regarding their clinical utility [5]. Together, these findings suggest that differences between physician training and institution-specific interpretation pathways may introduce variability in clinical applications and increase cognitive burden for providers. In time-sensitive settings, such variability may translate into delays in interpretation and reduce the efficiency with which VHA results are applied in practice.

This relationship between cognitive burden and efficiency has been observed in other complex clinical systems. A cross-sectional study evaluating electronic health record (EHR) use demonstrated that increased cognitive workload and fatigue were associated with reduced efficiency, including longer task completion times and increased burden with EHR interaction [6]. These findings highlight how complexity in clinical interfaces may impair workflow efficiency and delay action. Although VHAs differ from EHR systems, the requirement for rapid integration of complex, multi-parameter data suggests that similar usability challenges may arise when interpretation frameworks are not standardized.

Efforts to establish structured training frameworks rooted in standardized VHA-guided algorithms may help reduce variability in clinician understanding and improve familiarity, thereby facilitating more efficient clinical decision-making. A recent study evaluating iTEST, a structured, rule-based clinical decision-support tool, demonstrated that standardizing the interpretation of complex EHR data significantly reduced cognitive workload and improved efficiency, including shorter task completion times and enhanced usability compared with conventional workflows [7]. These findings underscore the importance of establishing structured frameworks to reduce cognitive burden and minimize clinical delay. In contrast, VHA-guided care often relies on institution-specific algorithms and heterogeneous training approaches, placing greater interpretive burden on individual clinicians and potentially contributing to delays in overall time to action. Furthermore, variability may arise from selective utilization of VHA parameters within these algorithms. Effective training frameworks should therefore emphasize not only interpretation of individual VHA metrics but also the collective integration of complementary parameters and assays within algorithmic approaches.

We encourage further investigation into optimized training strategies for VHA interpretation, with the goal of identifying approaches that improve efficiency and reduce cognitive burden. Subsequent efforts toward standardization of training and interpretive frameworks may be critical to enhancing clinician performance and reducing variability in efficient care delivery. Such efforts may ultimately help ensure that the hypothesized speed advantage of VHAs is reflected not only by device physiology but is also supported by effective training frameworks that enable timely and confident decision-making.

## Data Availability

No new data were created or analyzed in this study. Data sharing is not applicable to this article.

## Abbreviations

The following abbreviations are used in this manuscript:

VHA	Viscoelastic Hemostatic Assay
TIC	Trauma-Induced Coagulopathy
CCT	Conventional Coagulation Test
EHR	Electronic Health Record
